# Can Bodybuilding Peak Week Manipulations Favorably Affect Muscle Size, Subcutaneous Thickness, and Related Body Composition Variables? A Case Study

**DOI:** 10.3390/sports10070106

**Published:** 2022-07-05

**Authors:** Christopher Barakat, Guillermo Escalante, Scott W. Stevenson, Joshua T. Bradshaw, Andrew Barsuhn, Grant M. Tinsley, Joseph Walters

**Affiliations:** 1 Health Sciences and Human Performance Department, The University of Tampa, Tampa, FL 33606, USA; joshtbrad@competitivebreed.com (J.T.B.); abarsuhn@ut.edu (A.B.); jwalters@ut.edu (J.W.); 2 Competitive Breed LLC., Lutz, FL 33558, USA; 3 Department of Kinesiology, California State University, San Bernardino, CA 92407, USA; gescalan@csusb.edu; 4 Integrative Bodybuilding LLC., Tampa, FL 33617, USA; scottwstevensonme@me.com; 5 Department of Kinesiology and Sport Management, Texas Tech University, Lubbock, TX 79409, USA; grant.tinsley@ttu.edu

**Keywords:** carbohydrate loading, muscle thickness, subcutaneous thickness, contest preparation, physique athlete

## Abstract

Background: The purpose of this case study was to implement an evidence-based dietary approach to peaking for a bodybuilding competition and monitor its impact on body composition, muscle thickness (MT), intra-to-extra-cellular fluid shifts, subcutaneous thickness (ST), and hydration status. Secondarily, to document any adverse events of this peak week approach in a small, controlled setting. Methods Dietary practices were recorded, and laboratory testing was conducted throughout peak week, including competition morning. Assessments included: dual-energy X-ray absorptiometry (DEXA) for body composition, B-mode ultrasound for MT and ST, bioimpedance spectroscopy (BIS) for total body water (TBW)/intracellular water (ICW)/extracellular water (ECW), and raw BIS data (i.e., resistance, reactance, and phase angle), urine specific gravity (USG) for hydration status, and subjective fullness. Sequential dietary manipulations were made (i.e., CHO depletion/fat loading, CHO/water loading, and a refinement phase) with specific physiological goals. This was reflected in changes observed across all assessments throughout the peak week. Results: From the carbohydrate-depleted state (three days out) to competition day, we observed increases in lean body mass, MT, TBW (primarily ICW), and subjective fullness. Kendall’s Tau B revealed a strong relationship between carbohydrate intake and ∑MT (τ = 0.733, *p* = 0.056). Additionally, novel ST data demonstrated a 10% reduction for the summation of all seven sites, with some drastic changes in specific regions (e.g., −43% for triceps ST) from three days out to competition day. Conclusions: These data suggest that the prototypical goals of bodybuilders’ peak week (i.e., increasing muscle fullness, decreasing subcutaneous thickness) to enhance their aesthetics/muscularity presented can be achieved with a drug-free protocol involving dietary manipulations.

## 1. Introduction

Physique athletes compete in various divisions, including men’s bodybuilding, physique, and classic physique, as well as women’s physique, wellness, figure, bikini, and bodybuilding. Each category specifies varying levels of muscularity, symmetry, and leanness. Physique athletes generally periodize their training and nutrition based on which phase they are in and their proximity to competition [1,2,3]. Their ‘off-season’ phase is an extended period of time primarily focused on maximizing muscle hypertrophy through progressive resistance training in a hypercaloric state to support growth and recovery [1]. Whereas the ‘contest prep’ phase (often 8–24+ weeks) is primarily focused on reducing body fat to extreme levels for competition [2,4,5]. Despite the majority of the work and preparation taking place over a long period of time well before competition day itself, the final phase, ‘peak week’, can certainly improve or hinder the physique athlete’s aesthetics [4,5,6].

Physique athletes have, accordingly, been reported to implement various strategies to “peak” their body for competition day to enhance their aesthetics [4,7,8,9]. Since they are judged primarily on muscularity, symmetry, and “conditioning” (low body-fat levels), physique athletes often manipulate their exercise regimen, macronutrient intakes, fluid consumption, micronutrient balance, and dietary supplements throughout a contest preparation phase [2,5,9,10]. During the final week leading up to a competition (i.e., peak week) common practices such as carbohydrate depleting/loading, water loading/tapering, and others, have been reported in the literature with the intent of improving body composition, maximizing muscle glycogen and cell volume, minimizing subcutaneous water, and reducing the risk of abdominal bloating [4,7,9,11,12,13].

Research on the efficacy and safety of peak week protocols often employed by physique athletes is scarce even though it is commonly implemented. One study interviewed seven male bodybuilders during various phases of competition and reported that ~85% (six out of seven) competitors used a carbohydrate-loading strategy in an attempt to increase muscle glycogen content [9]. Additionally, 100% of competitors reported manipulating water intake while ~43% (three out of seven) simultaneously altered sodium intake in an attempt to create a leaner look [9]. In a larger study of 81 male and female bodybuilders, investigators utilized a survey to inquire about commonly utilized peaking interventions [7]. Similar to the results from the smaller sample of bodybuilders in the previously discussed study, investigators reported that ~93.8% of participants (76 out of 81) utilized a peak week strategy prior to competing [7]. Specifically, manipulation of carbohydrates, water, and/or sodium were most commonly used by competitors [7].

Although select observational data have highlighted some of the common practices physique athletes implement, even fewer studies have investigated how these practices are having an impact on physiological outcomes to determine their effectiveness. One case study of a natural bodybuilder, which utilized ultrasonography to assess muscle thickness, observed that two consecutive days of carbohydrate loading (~450 g/day, 5.1 g/kg) led to a ~5% increase of the biceps/triceps thickness and a ~2% gain in the quadriceps [5]. Similar findings were reported by Moraes et al. [11], in which muscle thickness of the elbow flexors and extensors increased by ~3% in competitors that implemented a carbohydrate-loading scheme. These findings are generally explained by the fact that each gram of glycogen requires ~3–4 g of water [14], thus, the carbohydrates consumed are increasing skeletal muscle volume. It has been theorized that this practice may have an impact on the intracellular-to-extracellular fluid shifts and carbohydrate loading may pull extracellular (including subcutaneous) water into the muscle, resulting in greater muscle size (“fullness”) while reducing subcutaneous thickness, thereby enhancing the visual delineation of individual muscles (commonly referred to “conditioning”) [5].

Until recently, there were a lack of objective data demonstrating whether bodybuilders were successful at manipulating the intracellular-to-extracellular water ratio to enhance their physique and peak for the competition. A recent pilot study [6] reported that physique athletes were able to successfully alter fluid from the extracellular to the intracellular compartment the final day of peak week. Their bioimpedance analysis revealed a ~1.5 L increase in intracellular water, a ~2.6 L decrease in extracellular water, which resulted in large and favorable increases to the intra-to-extra-cellular water ratio (1.60 to 1.92). Theoretically, this should enhance the athletes’ visual appearance by increasing muscle ‘fullness’ and ‘tightness’ by decreasing fluid underneath the skin. However, no data was presented regarding what practices (e.g., nutritional, water, or supplemental) were implemented to achieve the desired physiological outcomes observed.

Additionally, many bodybuilders seek to enhance their peak week physique by manipulating water intake [7,15], but the risk-to-reward ratio is not well understood. Recent observational data from members of our group (currently in revision) [10] demonstrated that water manipulation implemented by physique athletes led to significant or severe dehydration (urine specific gravity (USG) 1.021–1.030) on competition day in ~62% of the athletes in the study. It is unknown whether these practices are successful at achieving the objective goal (i.e., reducing subcutaneous water/thickness) or if it is leading to reduced muscle volume/size, the ability to achieve a “pump” (i.e., myocellular swelling/hyperemia via resistance training) backstage, and increasing health risks. Interestingly, data from Mitchell et al. [9] reported the competitors they investigated unanimously chose to abandon water manipulation practices in hindsight.

In 2021, members of our group co-authored a review article on an evidence-based approach to peak week recommendations for bodybuilders, discussing the potential benefits and risks of commonly used peaking strategies [4]. In addition to specifics related to training, dietary (i.e., carbohydrate, fat, protein, fiber, water, and electrolyte), and supplement intake during peak week, the review article provides general recommendations for bodybuilders to help readers develop individualized peak week strategies that coordinate all of the aforementioned variables. While the article emphasizes that these suggestions should not be considered concrete rules due to significant individual variability of how athletes may respond to the manipulation of these variables, it presents a logical and sequential approach to peak week with ample flexibility to make adjustments as needed.

Although the information presented in the physique athlete’s peak week review article [4] is based on the best available evidence reported in the literature, the efficacy and safety of the proposed general guidelines have not been investigated. As such, the purpose of this case study was to implement an evidence-based approach to peaking for a bodybuilding competition and to monitor how dietary practices affect body composition, muscle thickness, intracellular/extracellular fluid shifts, subcutaneous thickness, and hydration status. Secondarily, this case study allowed us to document any adverse events of an adaptation of this peak week approach on a small scale in a controlled setting, in particular as the primary investigator (CB) was the subject of the investigation.

## 2. Materials and Methods

### 2.1. Study Design and Setting

Assessments were performed on six days over an eight-day period at approximately the same time of day (8 a.m.). For the first five out of the six assessments, the subject reported to the laboratory after an overnight food and fluid fast (≥8-h), and consumed 473 mL of water upon waking, after collecting his first urination sample. The final testing session was conducted on the athlete’s competition morning (5 a.m.) after an overnight and fluid fast of just 5 h. The subject underwent the following assessments in the same order each day of data collection: urine specific gravity (USG) upon waking at home and in the laboratory, body mass (BM) (at home and in the laboratory), dual X-ray absorptiometry (DEXA), bioimpedance spectroscopy (BIS), subcutaneous thickness (ST) and muscle thickness (MT) via B-mode ultrasonography, arm circumference, and subjective muscle fullness.

### 2.2. Subject

The first author (CB) was the subject of this case study. He is a Middle Eastern-American (29 years old at the time of the investigation) professional natural bodybuilder who has been resistance training for 12 years. Data were collected over an eight-day period leading into the athlete’s second competition of the season (October 2021) as the athlete had been preparing for 24 weeks (May 2021 to October 2021) for this competition. The athlete competed in a weight-limit class (Open Middleweight Bodybuilding) and a height- and weight-divided class (Classic Physique A).

### 2.3. Procedures

#### 2.3.1. Dietary Intake and Fluid Consumption

The subject precisely tracked dietary intake by measuring each individual food source in grams, ounces, or ml via a food scale (Ozeri Pronto, ZK14-W) and inputted all nutrition data via the MyFitnessPal (Francisco Partners, San Francisco, CA, USA) phone application [16]. The subject also precisely tracked fluid intake while providing all dietary information in an Excel spreadsheet. Descriptive statistics were calculated to summarize dietary practices throughout the peak week.

#### 2.3.2. Body Composition, Bioimpedance, and Anthropometry

The subject’s height was measured using a mechanical stadiometer (DETECTO, Webb City, MO, USA), and total body mass was measured at two time points each assessment day, at home upon waking with a digital scale (Taylor, IL, USA), and in the laboratory before any other assessments. Body composition was assessed with multiple tools. This first assessment of fat mass (FM), lean body mass (LBM), and bone mineral content (BMC) was carried out via DEXA (Hologic, Inc., Marlborough, MA, USA) [17,18]. The subject’s positioning was in accordance with the manufacturer’s recommendations, lying supine with all body parts within the borders of the DEXA, and he remained still for the duration of the scan [17]. Thereafter, total body water (TBW), intracellular water (ICW), and extracellular water (ECW) were measured while the subject remained lying supine to avoid any fluid shifts that may occur with changing body positions. TBW, ICW, and ECW were measured using bioimpedance spectroscopy (BIS) (SFB-7; ImpediMed, Carlsbad, CA, USA), according to the manufacturers’ instructions [19]. The subject’s wrist, ankles, dorsal palm, and foot were cleaned with an alcohol wipe (70% isopropyl alcohol) and all regional body hair was removed. Dual-Tab Electrodes (L: 75 mm, W: 23 mm, ImpediMed, Carlsbad, CA, USA) were placed on the right side of the body for both the wrist and the ankle. Analysis of body composition was determined by the device with the subject remaining as still as possible. In addition to body fluid estimates, raw bioelectrical variables (resistance, reactance, and phase angle) were examined at the 50 kHz frequency.

Arm circumference measurements were obtained utilizing an anthropometric tape measure for the subject’s right arm, in a flexed position. The subject flexed the elbow joint and maximally contracted his biceps/triceps and a circumference measurement was obtained by a skilled researcher at the largest peak of the arm to the nearest 0.1 cm. Before recording the measurement, the researcher would ensure the tape was snug but did not compress the skin or muscle. This was repeated three times with the average value presented in the data.

#### 2.3.3. Subcutaneous Thickness and Muscle Thickness

Ultrasonography was employed to obtain ST by a skilled researcher on the right side of the body with the subject standing upright. Previous research specifically in athletes has demonstrated ultrasonography as a superior tool to measure subcutaneous adipose tissue thickness void of compression compared to skinfold thickness calipometry [20]. Two skilled researchers performed the assessments, one researcher on the probe (J.W.), the other on the monitor to freeze the image and take the measurement (J.B.), using a B-mode ultrasound device (HS40, Samsung Medison Co. Ltd., Seoul, Korea) and a linear probe (LA3-16AD, Samsung Medison Co. Ltd., Seoul, Korea) at frequencies between 5.3 and 6.7 Mhz. ST was assessed at these seven sites: chest, subscapular, triceps, midaxillary, suprailiac, abdomen, and thigh. One trained researcher applied a water-soluble transmission gel (Aquasonic 100; Parker Laboratories, Inc., Fairfield, NJ, USA) to the probe and then applied the probe to each measurement site with minimal pressure to ensure there was no compression of the subcutaneous tissue. When the second researcher was satisfied with the quality of the image, the image was saved, and ST dimensions were taken by measuring the distance from the top layer of the skin (superior dermis) to the most inferior portion of the subcutaneous adipose layer before the skeletal muscle (see Figure 2). To further ensure accuracy, three measurements were taken at each site and then averaged for the final value. The Jackson-Pollock seven-site formula was then used to estimate body-fat percentage [20]. Descriptive statistics were calculated to summarize changes in subcutaneous thickness at each region, the sum value, and body-fat percentage estimations.

Ultrasonography was also utilized to assess changes in MT throughout the peak week period. The same skilled researchers who performed the ST assessments via ultrasound also measured MT. Measurements were taken on the right side of the body at four different sites: (a) pectoralis major, (b) elbow flexors, (c) proximal anterior thigh, and (d) distal anterior thigh. For the pectoralis major, a distinguishable mark was made 2.54 cm superior-medial from the nipple on the sternal head. For the elbow flexors (brachialis and biceps brachii), measurements were taken by measuring the total distance from the lateral lip of the acromion processes to the lateral epicondyle of the radius and marking 37.5% of that total distance. A perpendicular mark was then made medially on the anterior surface of the bicep perpendicular to the 37.5% length measurement and parallel to the cubital fossa. For the anterior thigh assessments, femur length was measured in the sagittal plane from the greater trochanter to the lateral condyle. Marks were made at 40% (proximal) and 60% (distal) of the femur length in the frontal aspects of the thigh. Both anterior thigh images captured the total muscle thickness of the vastus intermedius and the rectus femoris. Thickness was measured from the periosteum of the rib, humerus, and femur, to the bottom of the muscle superficial fascia of the pectoralis major, bicep brachii, and rectus femoris for each respective site. Three measurements were taken at each point and the average was used for analysis.

#### 2.3.4. Subjective Fullness

To the authors’ best knowledge, subjective fullness has not been previously assessed in the literature. Subject fullness was attained purely based on the subject’s internal perception of muscle flatness versus fullness. Factors such as perceptual fullness at rest, fullness when muscles were flexed, how the muscles felt when palpated, the sensations of contraction, and how aggressively it felt pushing against the subcutaneous tissue/skin were taken into account. Zero would indicate maximal muscle flatness, and 10 would indicate maximal perceived fullness.

#### 2.3.5. Urine Specific Gravity

USG was obtained at two time points for each day of data collection: (a) upon waking, the first urination of the morning, and (b) upon arriving to the laboratory before any other assessments. The subject voided his bladder and provided a mid-stream urine sample for assessment of specific gravity of urine via refractometry (Tekcoplus Ltd., ATC Refractometer, Hong Kong, China).

### 2.4. Statistical Analyses

Data are reported in absolute value and per cent changes. Statistical process control was also used to determine trends in the data. Additionally, Kendall’s Tau-b correlation coefficient analysis was conducted to determine the relationship between variables (i.e., CHO intake and MT) in this single-subject design. The interpretation and use of Kendall’s Tau-b for single-subject design has been explained elsewhere [21]. The data were analyzed using Jamovi statistical software version 2.2.5 (Sydney, Australia) and Microsoft Excel version 2019 (Microsoft Corporation, Redmond, WA, USA). Significance was accepted at *p* ≤ 0.05.

## 3. Results

The subject competed on the final day of data collection and placed first in his classes (Open Middleweight Bodybuilding, Classic Physique Class A). The following data demonstrated the physiological adaptations that occurred throughout the peak week period in response to the practices employed.

### 3.1. Dietary Intake

Macronutrient and calorie intake varied throughout the peak week depending on the phase/objective the athlete was in (i.e., carb depletion, fat loading, and carb loading). Caloric intake ranged from 2206–3663 kcal (30.2–50.2 kcal/kg) throughout the peak week. Protein intake had the least variance throughout the week ~232 g/day, ~3.16 g/kg ranging from 195–285 g. During the fat-loading phase (i.e., six days out to four days out), dietary fat intake ranged from 86–132 g (114 g/day on avg, ~1.56 g/kg). Concomitantly, the athlete underwent a carbohydrate depletion phase, ranging from 73–88 g per day (~1.16 g/kg). During this period (i.e., six days out to four days out), water was held at a constant 6819 mL per day (93.4 mL/kg). Thereafter, carbohydrate loading began (three days out to one day out) and carbs were increased to ~578 g per day (7.9 g/kg), for two consecutive days. At this time, dietary fat intake was reduced to ~51 g/day (0.69 g/kg) and water was increased to ~10,229 mL per day (~140 mL/kg). One day out, the athlete consumed 81 g/fat (1.1 g/kg), 399 g carbohydrate (5.5 g/kg), and 228 g protein (3.1 g/kg), 3237 kcal (44 kcal/kg). Additionally, one day out, water was tapered slightly below baseline values to 5909 mL (80 mL/kg) (see Table 1). 

Micronutrient intake varied daily based on the food sources consumed and their nutritional value. However, sodium intake via salt added to each meal was held at a constant ~2300 mg/day throughout the majority of the peak week. From three days out to one day out, sodium intake via salt increased by ~33% to 3059 mg/day. Concomitantly, potassium intake was highest these two days (~6246 mg/day) and remained higher than baseline one day out from competition at 5862 mg, while total sodium intake was slightly tapered to 4227 mg (similar to baseline levels).

### 3.2. Body Composition

Bodyweight ranged from 72.45–73.55 kg throughout the peak week, with DEXA body-fat percentages ranging from 5.4–6.3%. The seven-site Jackson–Pollock formula was utilized via ultrasound measurements of the subcutaneous thickness and these calculations estimated body-fat percentages ranging from 3.6–4.2%.

DEXA outcomes demonstrated soft lean body mass increasing by 2% from its lowest value 65.56 kg (three days out, after the carbohydrate depletion phase), to its highest value 66.88 kg, one day out after the carbohydrate loading phase. Fat mass ranged from 3.97 kg to 4.63 kg via DEXA (see Table 2).

### 3.3. Muscle Thickness

Muscle thickness (MT) was assessed at four sites: the distal quadriceps, proximal quadriceps, pectoralis major, and elbow flexors (Table 3). MT of the distal quadriceps ranged from 5.39 to 5.70 mm. MT of the proximal quadriceps ranged from 6.52 to 6.78 mm. MT of the pectoralis major ranged from 2.53 to 2.81 mm. MT of the elbow flexors ranged from 3.93 to 4.10 mm.

The sum of all sites ranged from 18.43 to 19.01 mm, demonstrating a 3.15% increase from the three-day-out mark (after completing the carbohydrate depletion phase), to two days out (after one day of carbohydrate loading).

**Figure 1:**
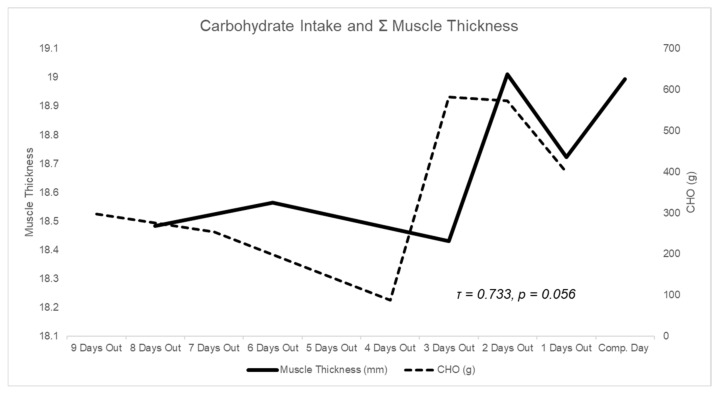
Daily carbohydrate intake during peak week and changes in summation of muscle thickness.

Exploratory Kendall’s rank correlation analyses indicated that ∑MT was positively associated with acute carbohydrate intake, the day before the assessment. (Τ = 0.733; *p* = 0.056) (Figure 1).

**Figure 2:**
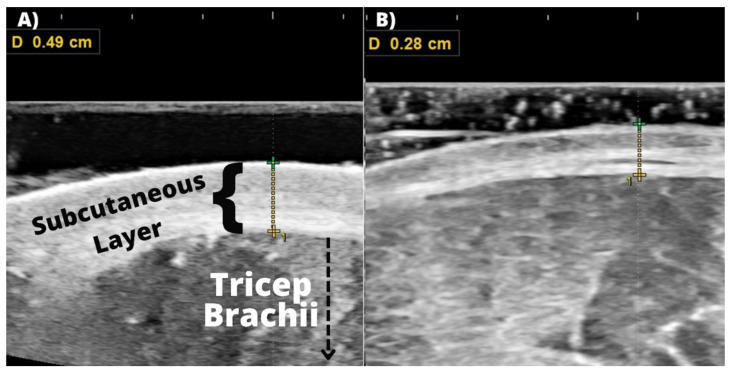
Subcutaneous Thickness. (**A**) Measurement of the subcutaneous layer superficial to the triceps brachii at three days out from the competition and (**B**) measurement of the subcutaneous layer superficial to the triceps brachii on competition morning.

### 3.4. Subcutaneous Thickness

The total sum for subcutaneous thickness varied by 8.92% throughout peak week, ranging from 29.6 to 32.5 mm (see Table 4). Some body parts presented highly variable changes in ST throughout the peak week while other regions were much more stable and consistent. For example, triceps ST reduced by ~43% from 4.9 mm three days out to 2.8 mm on competition day (see Figure 2). On the contrary, the ST of the chest decreased by ~10% from six days out (3.7 mm) to competition day (3.3 mm).

The chest, triceps, mid axillary, and thigh regions presented with their lowest ST value of the week on competition day. Additionally, the lowest ST sum value (29.6 mm) was observed on competition day.

### 3.5. Body Fluids

A slight, progressive decrease in ICW was observed from eight days out from competition through the day prior to competition. However, a 5.3% (1.75 L) increase was observed between one day prior to competition and the morning of competition. In contrast, ECW decreased slightly from eight days out from competition until three days out from competition, but was higher than baseline at two days out, one day out, and on the morning of competition. The largest increase in ECW occurred from three days out to two days out from competition, with a magnitude of increase of 6.2% (1.2 L). As expected, TBW followed a trend that exhibited characteristics of both ICW and ECW changes, with a slight decrease up until three days out from competition, followed by an increase to near baseline values one day out from competition, and the highest observed value on the morning of competition. The magnitude of increase in TBW from one day prior to the competition to the morning of the competition was 3.9% (2.1 L). The highest estimates of TBW, ICW, and ECW were all observed on the morning of competition.

**Figure 3:**
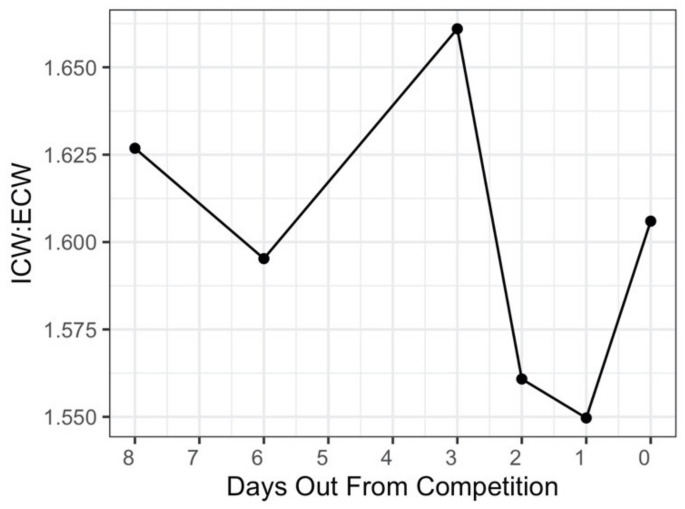
Intracellular Water to Extracellular Water Ratio from eight days out to competition day.

Over the course of peak week, the ICW:ECW ratio ranged from 1.55 to 1.66, with the lowest value observed one day prior to competition, and the highest value observed three days prior to competition (Figure 3). Between one day out and the day of competition, the ICW:ECW ratio increased from 1.55 to 1.61.

### 3.6. Raw Bioimpedance

**Figure 4:**
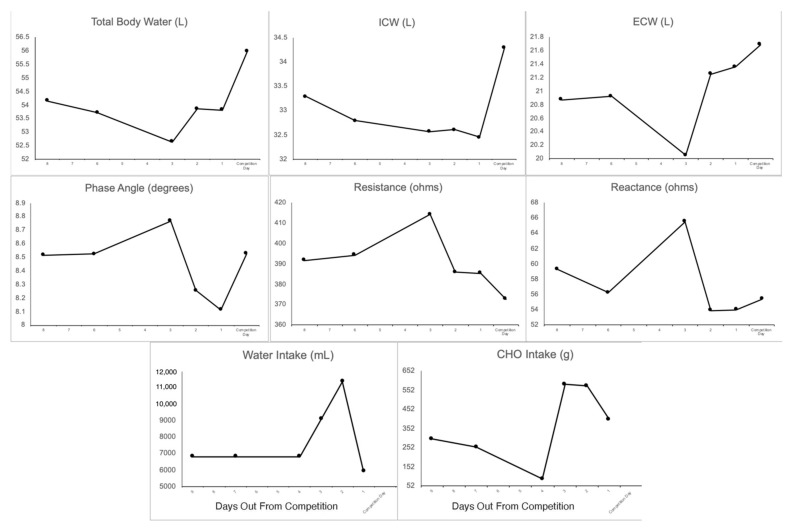
Total Body Water, Intracellular/Extracellular Water; Raw Bioimpedance: Phase angle, Resistance, Reactance, and dietary carbohydrate and water intake the day prior to BIS assessments.

Phase angle was stable at eight to six days out from competition, but increased to 2.9% above baseline three days prior to competition (Figure 4). The following day, two days out from competition, phase angle fell to 3.1% below baseline. An additional decline to 4.8% below baseline was observed the following day (one day out from competition). On competition day, phase angle increased and was nearly identical (D: 0.1%) to the value observed eight days prior to competition. Changes in resistance and reactance contributing to the observed changes in phase angle are displayed in Figure 4.

**Figure 5:**
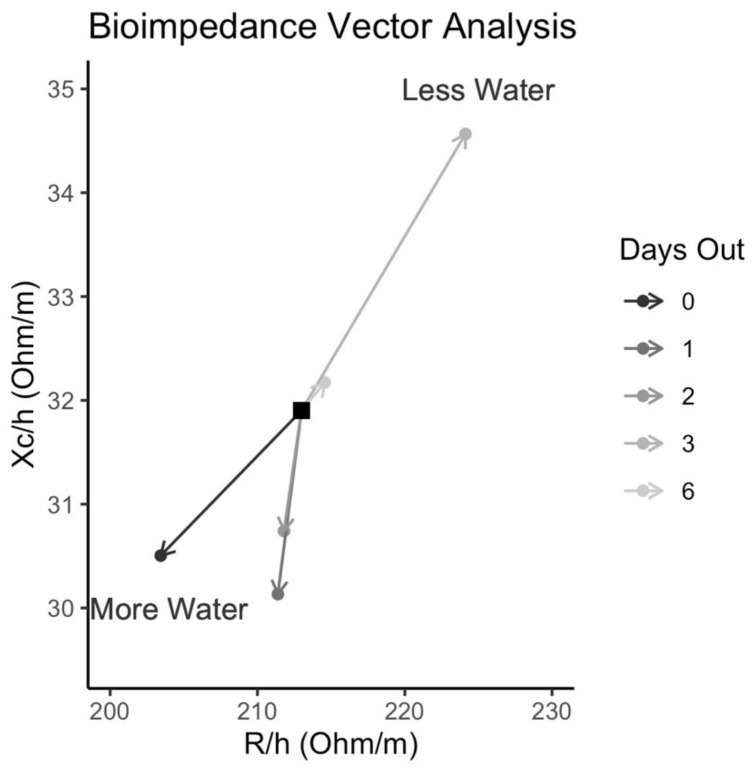
Bioimpedance Vector Analysis.

Bioimpedance vector analysis (BIVA) results are displayed in Figure 5. Relative to baseline (eight days out from competition), the bioimpedance vector migrated slightly to the upper right portion of the plot, indicative of less body water, at six days out from competition. This movement to the upper right portion of the plot notably increased from six days out to three days out from competition, indicating lower body water stores three days out. However, the bioimpedance vector migrated to the lower left portion of the plot, indicative of more body water than baseline, beginning at two days out from competition and lasting until the competition.

### 3.7. Hydration Status

Urine specific gravity and hydration status ranged from 1.01 to 1.018. Significant dehydration (1.021–1.030) nor severe dehydration (USG > 1.030) [22] was not reached despite the manipulation of water intake (see Table 5).

### 3.8. Subjective Muscless Fullness and Arm Circumference

Subjective skeletal muscle fullness was assessed from a 1–10 scale. One being as ‘flat’ as possible and 10 being as ‘full’ as possible. Subjective skeletal muscle fullness ranged from 1 out of 10, three days out, to 8 out of 10, on competition day (Table 6). 

Despite other assessments demonstrating changes to muscle mass, thickness, and intracellular water (i.e., soft LBM via DXA, US, BIS, respectively), arm-circumference measurements were unchanged from three days out to competition morning.

## 4. Discussion

The outcomes observed throughout this investigation provide further insight to the physiological impacts to specific bodybuilding peaking strategies. Some of the data are supported by previous studies [5,6,11] while other results demonstrate novel findings that have not been formally examined to date. Previous research has shown that bodybuilders implement a variety of peaking strategies with the aim of enhancing body composition, maximizing muscle size, and minimizing the interstitial space/subcutaneous thickness [4]; however, the success rate and physiological impact of these strategies are not well established. The dietary peaking practices employed in this investigation led to favorable outcomes in soft LBM, MT, ST, and ICW:ECW. We will discuss the sequential dietary changes and the outcomes studied in a phasic manner below in hopes to provide the readership with a comprehensive understanding of the impacts of the peak week practices employed.

The nutritional approach implemented throughout the peak week has various phases with specific goals. Those phases include the following: carbohydrate depletion/fat loading, carbohydrate/water loading, and a refinement period (i.e., one day out) to maintain ‘full’ muscle while increasing ‘tightness’/reducing ST.

The first phase, carbohydrate depletion/fat loading took place from six days out to four days out. During this period, carbohydrate intake was reduced to 1.1 g/kg per day, while water intake was held constant at 6.8 L (~78 mL/kg). Additionally, all carbohydrates consumed during this three-day period were from fibrous vegetable sources (e.g., Brussels sprouts, asparagus, etc.), not from starchy sources (e.g., oats, rice, or potato). This nutritional strategy was implemented as starchy sources (composed of many glucose molecules) are preferentially stored as muscle glycogen [23], whereas non-starchy sources (i.e., those high in fiber with a low glycemic index) [24,25], would theoretically not impair glycogen reduction. This carbohydrate-depletion practice is generally employed to potentiate the magnitude of glycogen super-compensation (and, thus, muscle volume) in the subsequent phase (i.e., phase two, carbohydrate loading) as previous literature has demonstrated its effectiveness [26,27]. Concomitantly, dietary fat intake was increased to 1.56 g/kg at this time for two primary purposes: to elevate intramuscular triglycerides and, thus, one aspect of myocellular volume [4,28], and to increase overall caloric intake near theoretical maintenance, rather than the athlete remaining in an energy-deficit/fat-loss focused phase (which constitutes the majority of a physique athlete’s total contest preparation). Previous literature has suggested that a fully ‘fat-loaded’ muscle may lead to increases > 1% in muscle volume [29]. Additionally, previous work suggests full intramuscular triglyceride restoration may be achieved when consuming 2 g/kg in a 24 h feeding window [30], which is on the order of the total fat consumed by the subject of the present study (~1.56 g/kg over the three-day window). Additionally, during this phase, protein intake was maintained ~3.45 g/kg per day, in accordance with the energy deficit of the preceding weeks of dieting [31,32,33].

During phase one, the carbohydrate-depletion phase, soft LBM reduced by ~1.32%, from 66.44 kg to 65.56 kg. This reduction in soft LBM coincided with a drop in TBW ~1 L/−1.8% (54 L to 53.01 L) while total body mass (BM) decreased by 0.81 kg/−1.1% (73.45 kg to 72.64 kg). This was further supported by changes in bioimpedance vector analysis (BIVA) as well, which involves visualization of changes in resistance and reactance relative to height and is used to estimate fluid and soft tissue changes [34]. Additionally, the lowest value for ∑MT was recorded after the depletion phase 18.4 mm, which is ~3% lower than the greatest ∑MT observed throughout the peak week. Furthermore, subjective fullness was scored a 1 out of 10, indicating the athlete felt ‘flat’ and depleted. Although muscle glycogen was not measured directly, these data suggest the low carbohydrate intake (1.1 g/kg) did result in the desired physiological outcome. These similar reductions in soft LBM, MT, TBW, BM, and subjective fullness seem predictable due to the fact that glycogen occupies ~2% of muscle cells [35] and each gram of glycogen is accompanied by 3–4 g of water [36]. Although intramuscular triglycerides were not directly measured (i.e., muscle biopsy), it is plausible to hypothesize that variables such as soft LBM, MT, and BM may have reduced even further during this phase if total dietary fat intake was lower and intramuscular triglycerides were not restored.

The second phase consisted of a carbohydrate load coupled with an increase in water and dietary salt. After depleting skeletal muscle glycogen, previous evidence suggests that a supercompensation effect is achievable where the muscle can store greater amounts of glycogen [36,37], thus, leading to greater intracellular fluid [14], muscle thickness [5,11], and lean body mass estimates [37]. From three days out to one day out, carbohydrate intake was increased to 7.9 g/kg per day, water was increased by ~49% to ~116 mL/kg, and sodium intake via added salt was increased by ~33%. Previous literature has demonstrated carbohydrate intakes ranging from 5–12 g/kg (with 10–12 g/kg being the most commonly suggested) can increase muscle thickness [5] and maximize glycogen stores [38,39]. Additionally, sodium–glucose dependent cotransporters have been shown to have a significant impact on the utilization of carbohydrates consumed by athletes [40,41,42]. Thus, to enhance glucose uptake and glycogen storage to maximize muscle fullness, ensuring adequate sodium intake in conjunction with carbohydrates, is a sensible approach [4,43]. Compared to previous observational data investigating the dietary practices of 21 male bodybuilders, this nutritional intake (for calories, carbs, and water) was much greater than the average of the cohort examined [10]. In that study [10], during the same time period (three days out to one day out) competitors, on average, consumed just 22.6 kcal/kg, 1.77 g carbohydrate per kg, and ~60 mL/kg of water; that is, ~52% fewer total calories, ~78% fewer carbs, and ~48% less water compared to the approach observed in this case study. Unfortunately, body composition and muscle thickness assessments were not performed so the physiological impacts of these nutritional approaches could not be compared. Although the peaking approach must be highly individualized due to large interindividual variability, these data suggest that many competitors may be undereating the amount of carbohydrates necessary to refill and maximize muscle glycogen [10]. The underconsumption of carbohydrates to maximize muscle glycogen has been repeatedly observed in other athletic populations [44,45], and more recent data suggest bodybuilders may also be implementing suboptimal dietary practices [10]. During this phase (i.e., carbohydrate/water loading), dietary fat intake was reduced by ~37% to 0.69 g/kg per day and dietary protein remained high but was also reduced to 2.84 g/kg per day.

The physiological impacts of these practices seem to produce favorable results in changes in soft LBM as well as MT. During phase two, the carbohydrate load, soft LBM increased by 2% from 65.56 to 66.88 kg. Similarly, ∑MT values also increased by ~1.6%, with notable differences dependent on the region. The distal and proximal quadriceps demonstrated the largest increase in MT (5.75% and 3.67%) in a 24 h period, from three days out (after completing the carbohydrate depletion) to two days out (after one day of carbohydrate loading). Previous research [5] has demonstrated similar results in the lower limbs where quadricep MT increased by ~2% after two consecutive days of consuming 450 g carbohydrates per day (~5 g/kg). However, that data also resulted in a ~5% increase in MT of the elbow flexors where we did not observe any changes at that site, nor in arm circumference. When considering the unchanged MT of the elbow flexors and arm circumference, coupled with the subjective muscle fullness assessment never exceeding an 8 out of 10, this data suggests that maximal muscle glycogenesis may not have been achieved. The magnitude of change in different muscle groups may suggest more carbohydrates could have been consumed during the loading phase. These positive changes to soft LBM and MT were also supported by the concomitant changes in TBW. TBW increased by 1.13 L (~2.13%) and, as previously mentioned, these data can be explained by the relationship between carbohydrate loading and water required for muscle glycogenesis [35,36]. Lastly, in conjunction with the increases in objective markers (i.e., soft LBM, MT, and TBW), subjective fullness increased from a 1 out of 10, to 7 out of 10 during this two-day, carbohydrate-loading phase.

During the final “refinement” phase, one day out from competition, the goal was to maintain muscle ‘fullness’ (i.e., soft LBM and MT), while enhancing the overall aesthetic by reducing ST to create a tighter and more ‘conditioned’ appearance. This would be accomplished by minimizing activity (training, posing, etc.) that would call upon the previously elevated glycogen and triglyceride stores, thus maintaining these components of muscle fullness, while manipulating water and sodium intake to reduce total body water, including interstitial, subcutaneous water. Nutritionally, total calories remained high (44 kcal/kg), with macronutrient intake being more balanced; fat intake was increased to 1.1 g/kg, carbohydrates were slightly decreased to 5.5 g/kg, and protein was slightly increased to 3.1 g/kg. Previous research has demonstrated that glycogen supercompensation can be maintained for at least three days with a moderate carbohydrate intake [36]. Additionally, data has suggested that carbohydrate loading phases can reduce intramuscular triglyceride levels [30,46], thus increasing dietary fat intake during this refinement phase may enhance muscle fullness and is a secondary goal [4]. Moreover, water intake was reduced by ~46% but remained relatively high at 80 mL/kg. From one day out to competition morning, ∑MT continued to increase by 1.44% (18.72 mm to 18.99 mm), with differing responses depending on the site. For example, the pectoralis major MT increased by ~8% (2.6 mm to 2.81 mm) in just 24 h, whereas the elbow flexor MT remained unchanged. Additionally, soft LBM was ~0.8% lower on competition day compared to one day out (66.88 to 66.32 kg). As all assessment methods have their limitations and standard error rates, this minor reduction in soft LBM should be taken with caution. We will expand on those potential limitations and specific details later in this discussion section.

Despite the dietary changes made one day out from competition, some variables presented differently than predicted. For example, total BM slightly increased (0.1 kg) and TBW increased by 2.1 L (54.14 to 56.24 L) despite reductions in carbohydrate intake (573 to 399 g) and a ~46% taper in water. Previous data that utilized a similar water load, yet a more aggressive taper yielded a significant reduction in BM (~3.2% on avg.) [47] due to an increase in diuresis. One factor that potentially explains our data is that competition day assessments occurred at 5:00 AM compared to all other assessments occurring at 8:00 AM and the subject sleeping just 3 h 21 min from one day out to competition morning [48], while averaging >6 h the previous days of data collection (see Supplementary Materials—OURA ring sleep data). Interestingly, ~83% of the increases in TBW were estimated to be stored as ICW (+1.75 L), which improved the ratio between ICW and ECW from one day out (1.55) to competition morning (1.61). Although it is difficult to explain these favorable acute changes, recent data from a cohort of 11 male bodybuilders demonstrated similar results [6]. Nunes et al. [6] demonstrated positive changes in ICW:ECW from one day out to competition day, however, they also observed reductions in TBW and ECW (which is what we hypothesized but did not observe). Unfortunately, the dietary practices employed by those 11 competitive bodybuilders was not reported so comparisons cannot be made. It is plausible to suspect that pharmaceutical diuretics may have been employed as these physique athletes were competing in an untested (not drug-free) show [6], and previous cohorts have reported their use [10,49,50]. Additionally, one potential reason for differences observed between TBW and ECW may be explained due to differences between bioelectrical impedance devices used and the assumptions made to estimate fluid volumes.

Recently, compelling arguments for the advantage of using raw bioimpedance metrics for the evaluation of hydration (rather than subsequent estimations of fluid volumes) have been presented [34,51]. Particularly given the likelihood that the physique competitor being assessed in the present study was dissimilar (i.e., limb circumference, relative soft LBM, body fat %, etc.) to the populations in which body-fluid volume prediction equations were developed [51,52], an examination of the raw bioelectrical data was warranted [17]. Perhaps, the most frequently used bioimpedance metric is the phase angle, which incorporates information about bioelectrical resistance and reactance to provide an indication of underlying cellular properties [53]. Similar to the findings of Nunes et al. [6], phase angle increased by ~0.3° from one day out to competition day. While the water intake the day prior to assessments could have also played a role in phase angle fluctuations, the implementation of an overnight food and fluid fast may have minimized the role of water intake per se the day prior as compared to the influence of glycogen stores and the associated water content (i.e., ICW).

Bioimpedance vector analysis (BIVA) incorporates changes in bioelectrical resistance (i.e., the opposition to current flow) and reactance (i.e., the capacitive properties of the cell membranes) and allows for visualization of changes in body fluids and other components. BIVA assessments indicated an initial decrease in body water, relative to the baseline assessment at eight days prior to competition, particularly at three days out from competition. This day also corresponded to the lowest estimates of TBW and ECW, as well as low values of ICW. Thereafter, a notable shift in the bioimpedance vector, indicative of increased body water, was observed beginning at two days prior to competition. This dramatic shift coincided with the large increase in carbohydrate intake previously described. While preliminary, these observations indicate the sensitivity and potential utility of raw bioelectrical variables, including phase angle and BIVA, for evaluating responses to dietary manipulations in the week preceding a physique competition. This data, in conjunction with the soft LBM, MT, and subjective fullness suggest that muscle fullness was successfully preserved and potentially enhanced from three days out to competition day. Additionally, novel findings were observed regarding ST that are likely affected by changes in body water and fluid shifts throughout the peak week.

To the authors’ best knowledge, no previous study has investigated acute changes to ST across consecutive days. During the peak week phase, we observed drastic changes in ST throughout the body and this data may provide notable insights to the practices implemented and the outcomes produced. Changes in ST were highly variable from region to region and day to day. However, the ∑ST ranged from 32.9 mm to 29.6 mm and this 10% decrease occurred in less than 48 h from two days out to competition morning. Moreover, some body parts, such as the triceps, demonstrated a 42% reduction in ST from three days out (4.9 mm) to competition morning (2.8 mm) (see Figure 2). Additionally, the quadriceps also demonstrated a ~22% reduction in ST, from three days out (5.8 mm) to competition morning (4.5 mm). These reductions in ST may be explained by the water demands of glycogenesis pulling fluid from the subcutaneous region [4,5], acute changes in water content of the adipocytes, and the intentional manipulation of fluid intake. Water content of adipocytes is highly variably ~6–36% [54], thus, changes in this tissue would be reflective in ST. Additionally, the water-manipulation strategy implemented has been shown to increase diuresis, urine output, and vasopressin [47], thus, it is plausible that water reduction occurred from the subcutaneous/interstitial space, resulting in lower ST. Assuming muscle glycogen was concomitantly maintained or increased (indirectly based on MT, soft LBM, and ICW), this would result in the peak week goals of achieving both greater muscle ‘fullness’ as well as ‘tightness’ by way of reducing ST. Furthermore, increases in muscle glycogen and ICW may have increased pressure between the skeletal muscle and skin, literally tightening the skin/muscle interface, causing ST to decrease at specific sites. However, some regions did not demonstrate reductions in ST and the hip was one region that actually displayed a ~35% increase in ST during this time period (three days out, 3.1 mm vs. competition morning, 4.2 mm). This may be due to the differences in muscle architecture, absolute size, thickness, function, and how deep or superficial the muscles in the region being assessed are (e.g., external obliques (hip) vs. quadriceps (anterior thigh)). These data demonstrate that water is held between the skin and skeletal muscle interface and suggest that visual changes/common qualitative terms used by bodybuilders, such as ‘watery’ versus ‘dry’ [4], can be observed/quantified.

Bodybuilders are notorious for manipulating and pushing the boundaries of hydration status [49,50,55], which is, lastly, an important variable to discuss. Besides manipulating dietary intake (i.e., macro/micronutrient, water), as previously mentioned, bodybuilders have reported using pharmaceutical diuretics [49,55] amidst evidence highlighting their dangers and associated health risks [4] and despite a lack of evidence supporting their intended effects in the context of bodybuilding. Recent observational data from members of our group demonstrated that ~62% of the physique athletes examined presented with significant or severe dehydration (USG 1.021–1.030) on competition day [10]. The goal in manipulating variables such as carbohydrates, water, sodium, and potassium is to achieve a ‘full and dry’ appearance [4]. In our case, although water intake was manipulated (i.e., loaded then tapered) with the intent to increase diuresis, enhance body composition, and body water distribution (ICW:ECW), neither significant dehydration (1.021–1.030) nor severe dehydration (USG > 1.030) [22] was observed using the peak week strategy employed here. Thus, our results illustrate a bodybuilding peaking method that can positively affect muscle size, soft LBM estimates, and ICW:ECW, and reduce ST without compromising health and safety.

This study helps bridge the gap between practices based on logical scientific theories that have been implemented by coaches and athletes for decades and their application’s physiological efficacy. These data are important as it gives us objective insight in a field and population demographic with such limited information. Although our study provides detailed assessments of changes in body composition in response to dietary practices and novel information regarding acute changes to ST, there are some inherent limitations that must be considered. Firstly, although testing conditions were standardized to the researcher’s best ability, there are standard error rates for all modes of body composition assessments [18,56,57]. Unfortunately, some of the standard error rates may equate, or even be greater than the actual changes we are expecting, to capture from the assessment tool. Additionally, some assessments are functioning under specific assumptions (e.g., tissue density, hydration status, etc.) and were developed in populations dissimilar to the bodybuilder examined in this case study [17,51,52]. For example, the bioelectrical spectroscopy device we utilized to estimate TBW, ICW, and ECW assumes specific ‘cylinder circumferences’ (i.e., upper arm and thigh) and other validation formulae for athletic populations [51] were extrapolated from a sample of younger males (~22 y.o.) with much higher bodyfat levels (~15%) than a contest lean bodybuilder (3.6–6.3%). Thus, as previously mentioned, this warranted further investigation and reporting of the raw bioimpedance values (i.e., resistance and reactance). Additionally, another limitation to consider is the inherent variance and actual macro and micronutrient [58,59] content of the food consumed compared to the estimated values logged through the MyFitnessPal application. Some food sources/products did not contain values for sodium and/or potassium but were all logged as reported. Lastly, the main limitation with a case study is that the outcomes have limited extrapolation to a specific population. Although the protocol applied here is rooted in physiological science and well-known techniques among bodybuilding competitors, the extent to which other athletes would respond similarly cannot be determined. Thus, more research is warranted in the competitive bodybuilding realm so coaches and athletes can develop safe and effective peak week strategies.

## 5. Conclusions

This case study supports the effectiveness of coordinated dietary manipulation to achieve the goals of a bodybuilding ‘peak week’ of increasing muscle ‘fullness’, quantified here as muscle thickness, and ‘drying out’, as seen in a reduction in subcutaneous thickness. These results are supported by the shift of body water toward the intracellular space, increasing muscularity and reduced subcutaneous fluid. Additionally, no adverse effects were noted, and urine specific gravity did not suggest dehydration in this subject. These protocols and data reported here provide a basis for further research toward developing both safe and effective methods bodybuilders can employ to enhance on-stage presentation.

## Figures and Tables

**Table 1:** Macronutrient, Water, Sodium, and Potassium.

Nutrition (The Day before Assessments)									
Days Out	Fat (g)	Carb (g)	Pro (g)	Kcal	Water (mL)	Na+ via Added Salt (mg)	Na+ from Food (mg)	∑ Na+ (mg)	K+ from Food (mg)
Nine	39	297	249	2535	6819	2300	1644	3944	1775
Seven	53	254	207	2321	6819	2300	1011	3311	1567
Six	132	88	217	2408	6819	2300	2647	4947	2103
Five	86	73	285	2206	6819	2300	5227	7527	2153
Four	125	88	253	2489	6819	2300	2926	5226	3375
Three	46	582	195	3522	9092	3059	1384	4443	6522
Two	55	573	219	3663	11,365	3059	2242	5301	5971
One	81	399	228	3237	5909	2300	1927	4227	5862

**Table 2:** Body Composition.

	DEXA						JP-7 via US
	Body Mass (kg)	Body Fat %	FM (kg)	FFM (kg)	BMC (kg)	Soft LBM (kg)	Body Fat %
Eight Days Out	72.45	5.5	3.99	68.47	2.591	65.88	n/a
Six Days Out	73.45	6.0	4.41	69.05	2.606	66.44	3.6
Three Days Out	72.64	6.2	4.50	68.13	2.573	65.56	4.1
Two Days Out	73.18	5.6	4.10	69.08	2.610	66.47	4.2
One Day Out	73.45	5.4	3.97	69.49	2.608	66.88	3.8
Competition Day	73.55	6.3	4.63	68.91	2.591	66.32	3.6

**Table 3:** Muscle Thickness (mm) via Ultrasound.

	Distal Quad	Proximal Quad	Chest	Elbow Flexors	SUM
Eight Days Out	5.42	6.55	2.58	3.93	18.48
Six Days Out	5.39	6.52	2.56	4.10	18.56
Three Days Out	5.39	6.54	2.53	3.96	18.43
Two Days Out	5.70	6.78	2.53	4.00	19.01
One Day Out	5.52	6.66	2.60	3.94	18.72
Competition Day	5.60	6.63	2.81	3.95	18.99

**Table 4:** Subcutaneous Thickness (via B-mode Ultrasound).

	Chest (mm)	SubScap (mm)	Triceps (mm)	MidAx (mm)	Hip (SA) (mm)	Ab (mm)	Thigh (mm)	Total Sum
Six Days Out	3.7	5.4	3.0	4.3	3.8	5.0	4.6	29.8
Three Days Out	3.5	5.8	4.9	5.2	3.1	4.2	5.8	32.5
Two Days Out	3.5	6.0	3.5	5.0	3.8	6.0	5.1	32.9
One Day Out	3.4	5.8	3.1	4.1	3.8	5.6	4.8	30.6
Competition Day	3.3	5.9	2.8	4.0	4.2	4.9	4.5	29.6

**Table 5:** Urine Specific Gravity.

	Refractometer	
Eight Days Out	Upon Waking	1.012
	Pre-Assessment	1.018
Six Days Out	Upon Waking	1.010
	Pre-Assessment	1.012
Three Days Out	Upon Waking	1.012
	Pre-Assessment	1.011
Two Days Out	Upon Waking	n/a
	Pre-Assessment	1.012
One Day Out	Upon Waking	1.010
	Pre-Assessment	1.010
Competition Day	Upon Waking	1.018
	Pre-Assessment	1.012

**Table 6:** Subjective Muscle Fullness and Arm Circumference.

	Subjective Muscle Fullness (1–10)	R Arm Circumference (cm)
Three Days Out	1	40.5
Two Days Out	4.5	40.5
One Day Out	7	40.5
Competition Day	8	40.5

## Data Availability

All data collected and/or analyzed during this study are included in this published article and its Supplementary Information Files.

## Supplementary Materials

The following supporting information can be downloaded at: https://www.mdpi.com/article/10.3390/sports10070106/s1, Supplementary Materials File S1: All Raw Data Spreadsheet + Oura Ring Sleep Data; Supplementary Materials File S2: Subject Competition Photo.
