# Supplementary figures and images for: Can Bodybuilding Peak Week Manipulations Favorably Affect Muscle Size, Subcutaneous Thickness, and Related Body Composition Variables? A Case Study
Source: Sports (Basel). 2022 Jul 5;10(7):106. doi: 10.3390/sports10070106 (PMC9321665; doi:10.3390/sports10070106)

OURA RING SLEEP DATA)

SM2: SUBJECT/ATHLETE/AUTHOR PHOTO

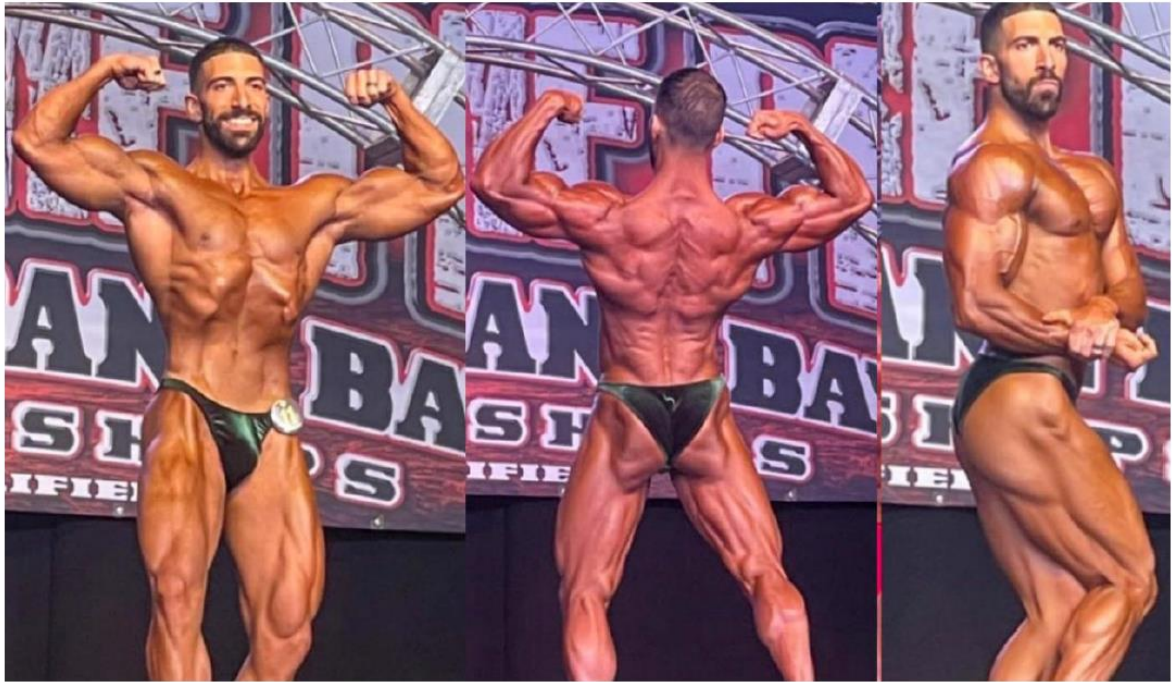

Supplement: Supplementary file 1 [file sports-10-00106-s001.zip › SM2.pdf]
